# Antihepatoma Activity of *Artocarpus communis* Is Higher in Fractions with High Artocarpin Content

**DOI:** 10.1155/2014/978525

**Published:** 2014-07-14

**Authors:** Cheng-Wei Tzeng, Feng-Lin Yen, Liang-Tzung Lin, Chiang-Wen Lee, Ming-Hong Yen, Wen-Sheng Tzeng, Chun-Ching Lin

**Affiliations:** ^1^Graduate Institute of Natural Products, College of Pharmacy, Kaohsiung Medical University, No. 100 Shih-Chuan 1st Road, Kaohsiung 80708, Taiwan; ^2^Department of Fragrance and Cosmetic Science, College of Pharmacy, Kaohsiung Medical University, No. 100 Shih-Chuan 1st Road, Kaohsiung 80708, Taiwan; ^3^Institute of Biomedical Sciences, Sun Yat-Sen University, No. 70 Lienhai Road, Kaohsiung 80424, Taiwan; ^4^Department of Microbiology and Immunology, School of Medicine, College of Medicine, Taipei Medical University, No. 250 Wu-Hsing Street, Taipei 11031, Taiwan; ^5^Department of Nursing, Division of Basic Medical Sciences, and Chronic Diseases and Health Promotion Research Center, Chang Gung Institute of Technology, No. 2 Chia-pu Road, West Section, Chiayi 61363, Taiwan; ^6^Research Center for Industry of Human Ecology, Chang Gung University of Science and Technology, No. 261 Wen-Hwa 1st Road, Taoyuan 33303, Taiwan; ^7^School of Pharmacy, College of Pharmacy, Kaohsiung Medical University, No. 100 Shih-Chuan 1st Road, Kaohsiung 80708, Taiwan; ^8^Department of Medical Imaging, Chi Mei Medical Center, No. 901 Zhonghua Road, Tainan 71004, Taiwan; ^9^Department of Medical Imaging and Radiological Science, College of Health Sciences, Central Taiwan University of Science and Technology, No. 666 Buzih Road, Taichung 40601, Taiwan

## Abstract

Extracts from natural plants have been used in traditional medicine for many centuries worldwide. *Artocarpus communis* is one such plant that has been used to treat liver cirrhosis, hypertension, and diabetes. To our knowledge, this study is the first to investigate the antihepatoma activity of *A. communis* toward HepG2 and PLC/PRF/5 cells and the first to explore the relationship between antihepatoma activity and the active compound artocarpin content in different fractions of *A. communis*. *A. communis* methanol extract and fractions induced dose-dependent reduction of tumor cell viability. DNA laddering analysis revealed that *A. communis* extract and fractions did not induce apoptosis in HepG2 and PLC/PRF/5 cells. Instead, acridine orange staining revealed that *A. communis* triggered autophagic cell death in a dose-dependent manner. The antihepatoma activity of *A. communis* is attributable to artocarpin. The fractions with the highest artocarpin content were also the fractions with the highest antihepatoma activity in the following order: dichloromethane fraction > methanol extract > ethyl acetate fraction > *n*-butanol fraction > *n*-hexane fraction. Taken together, *A. communis* showed antihepatoma activity through autophagic cell death. The effect was related to artocarpin content. Artocarpin could be considered an indicator of the anticancer potential of *A. communis* extract.

## 1. Introduction

Plant extracts have been applied in traditional medicine for curing and preventing diseases over at least the past 5,000 years. The World Health Organization estimates that more than 80% of the population of developed countries primarily use traditional herbal remedies to treat a variety of diseases [1]. More than 80,000 plants have a recognized medical use. For instance, PubMed database retrieves more than 35,000 papers on the anticancer activities of plants and on the clinical application of their active ingredients, for example, those of curcumin [2] and* Azadirachta indica* L [3].

Programmed cell death is a complex process referred to as any form of cell death mediated by an intracellular program, which is classically known as apoptosis and autophagy [4, 5]. Apoptosis, known as type I programmed cell death, plays a major role in multicellular organisms. Apoptosis is a rapid and irreversible process characterized by chromatin condensation, nuclear breakdown, and DNA fragmentation [6]. The entire process is controlled by several intrinsic and extrinsic pathways, such as the caspase activation cascade [7]. On the other hand, autophagy, known as type II programmed cell death, is characterized by the presence of double-membrane autophagosomes and the activation of autophagy-related (*Atg*) genes that control the formation of autophagic vesicles [8]. Autophagy is discriminated from apoptosis by its distinctive nonapoptotic morphology (autophagosomes) [9] and caspase-independent mechanisms [10]. Many cancer studies have focused on apoptotic and autophagic mechanisms for elucidating the anticancer activity of plants and their active ingredients.

*Artocarpus communis*, which belongs to Moraceae family, is wildly distributed in the tropical and subtropical regions of Asia.* A. communis* offers substantial economic value as a multipurpose crop that produces timber and fruits throughout the entire year. Its extraordinary medicinal value, such as in the treatment of diabetes, malarial fever, and diarrhea, has long been recognized in Southeast Asia [11]. Previous researches have shown that* A. communis* contains substances such as flavonoids, triterpenoids, steroids, stilbenes, and fatty acids [11]. Isoprenyl flavones have also been extracted from plants of the* Artocarpus* genus in Taiwan. One such isoprenylated flavone is artocarpin, a compound with many bioactivities such as antioxidation [12] and antimelanoma [13] activities, attenuation of ultraviolet* B*-induced skin damage [14], and inhibition of melanin biosynthesis [15]. The anticancer activity of* A. communis* and artocarpin toward several cancer cell lines was confirmed in previous studies [13, 16, 17], but the effect of* A. communis* and artocarpin on hepatoma HepG2 and PLC/PRF/5 cell lines has not been reported yet. In addition, it is unknown whether there is any relationship between the anticancer activity and the content of artocarpin.

In this study, organic solvent fractions of* A. communis* heartwood were investigated for their antihepatoma activity by using a cell viability assay. High-performance liquid chromatography with an ultraviolet detector (HPLC-UV) was used to quantify the active compound artocarpin in different fractions of* A. communis*. Results showed that* A. communis* fractions exhibited antihepatoma activity in an artocarpin dose-dependent manner. Artocarpin could be regarded as an indicator for the anticancer potential of* A. communis* extract.

## 2. Material and Methods

### 2.1. Chemicals and Reagents

1-(4,5-Dimethylthiazol-2-yl)-3,5-diphenyl tetrazolium bromide (MTT) and dimethyl sulfoxide (DMSO) were purchased from Sigma-Aldrich Chemicals Co. (St. Louis, MO). Dulbecco's modified Eagle's medium (DMEM), penicillin G, streptomycin, and amphotericin B were purchased from GIBCO BRL (Gaithersburg, MD). All other chemical reagents were of analytical grade.

### 2.2. Plant Material and Extraction

The heartwoods of* A. communis* were purchased from Tainan District Agricultural Research and Extension Station. The authenticity of the plant species was confirmed by a pharmacognosist (Dr. Ming Hong Yen). Two kilograms of heartwood of* A. communis* was sliced and immersed in a container of 20 L of methanol at room temperature for 2 weeks. The methanol extract (AM) was filtrated and concentrated by rotary vacuum evaporation and then lyophilized with a freeze-dryer. The crude methanol extract was resuspended in 1500 mL of water and then consecutively partitioned with the equivalent volume of* n*-hexane (AH), dichloromethane (AD), ethyl acetate (AE), and* n*-butanol (AB). The organic solvent of each extract was concentrated and removed by rotary vacuum evaporation. The final extracts were lyophilized with a freeze-dryer and the powders were collected and stored at −20°C until use.

### 2.3. Measurement of the Artocarpin Content by HPLC

The HPLC system (Hitachi, Japan) consisted of a pump (L-7000), an autosampler (L-7200), an L-7420 UV-vis detector, and a D-7000 interface module. All the test samples were analyzed on a Lichro-CART 250-4 Purospher STAR RP-18e (250 × 4.6 mm i.d., 5 *μ*m) column, and the temperature was kept constant at room temperature. The HPLC system mobile phase was composed of methanol and water (9 : 1), the flow rate was set at 1 mL/min, and the wavelength of detection was 282 nm. The artocarpin content in the methanol extract and fractions of* A. communis* was determined by comparing the retention time with the artocarpin standard.

### 2.4. Cell Lines and Culture

Human hepatoma cell lines HepG2 and PLC/PRF/5 were obtained from Bioresource Collection and Research Center (Hsinchu, Taiwan). Cells were cultured in DMEM supplemented with 10% fetal bovine serum, 100 U/mL of penicillin G, 100 *μ*g/mL of streptomycin, and 0.25 *μ*g/mL of amphotericin B. The cells were maintained under standard cell culture conditions at 37°C and 5% CO_2_ in a humidified incubator.

### 2.5. Cell Viability Assay

To determine cell viability, a microculture tetrazolium assay was performed according to the method previously described by Moghaddam et al. [18] with minor modifications. Quantitative measurement of viable cells was provided by MTT assay by determining the amount of formazan crystals produced by metabolically active cells. Briefly, the cells were seeded into a 96-well plate (1 × 10^4^ cells per well). After 24 h of incubation, the culture medium was replaced with fresh medium containing* A. communis* fractions (AM, AD, AE, AB, and AH) and artocarpin, and the cells were incubated for further 24 h. DMSO (1%, v/v) was used as a negative control. MTT reagent was added to each well. The plate was incubated for further 4 h, and thereafter the medium was discarded and the formazan crystals were dissolved in 150 *μ*L of DMSO. The absorbance of cells was measured at 550 nm. The data are presented as percentage of viable cells (%).

### 2.6. Clonogenic Assay

A clonogenic assay was performed as described [19] with some modifications. Briefly, HepG2 and PLC/PRF/5 cells were seeded into six-well plates at a density of 10^3^ cells per well. Twenty-four hours after seeding, the cells were treated with various concentrations of* A. communis* extract and fractions for 9 days. Drugs were replaced when medium was refreshed. Plates were then washed with PBS, and cells were fixed in methanol and stained with 0.1% Coomassie blue (Bio-Rad, Hercules, CA) in 30% methanol and 10% acetic acid. Images were captured by a CCD camera.

### 2.7. DNA Laddering Assay

PLC/PRF/5 and HepG2 hepatoma cells were collected by trypsinization and their DNA was isolated by using the ApopLadder Ex Kit (Takara Bio Inc., Otsu, Japan) according to the manufacturer's instructions. Isolated DNA was dissolved in TE buffer (10 mM Tris-HCl, 1 mM EDTA, pH 7.5) and then diluted in 6x loading buffer (5 : 1 in a total volume of 6–15 *μ*L). The mixture was subjected to electrophoresis using a 2% agarose gel and Tris-borate-EDTA buffer at 50 V. The DNA fragmentation pattern was visualized with a UV transilluminator.

### 2.8. Acridine Orange Staining

Acridine orange (Millipore, Billerica, MA) was added to* A. communis* fractions and artocarpin treated cells at a final concentration of 5 *μ*g/mL for a period of 15 min. Pictures were obtained with a fluorescence microscope (Nikon ECLIPSE TS100) equipped with a mercury 100 W lamp, 490 nm band-pass blue excitation filters, a 500 nm dichroic mirror, and a 515 nm long pass barrier filter.

### 2.9. Statistical Analysis

All data are expressed as means ± standard deviations of the indicated number of experiments. One-way analysis of variance (ANOVA) was used to evaluate the data and a post hoc test of Scheffe's method was used to calculate statistical significance using SPSS software. *P* < 0.05 was considered statistically significant. All assays were performed in at least three independent experiments.

## 3. Results

### 3.1. Artocarpin Content of* A. communis* Methanol Extract and Its Fractions

**Figure 1:**
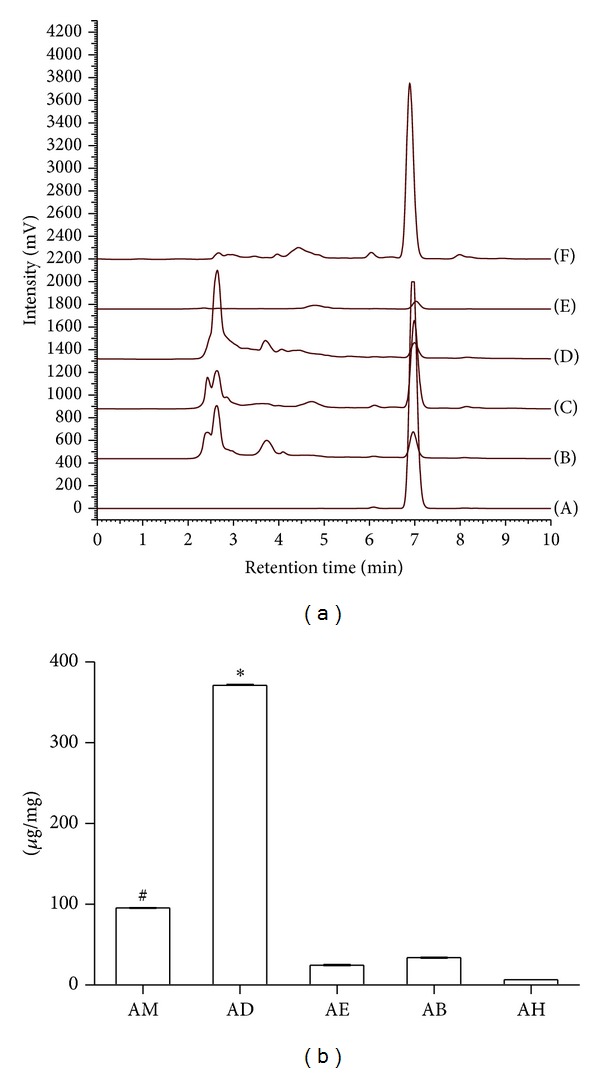
The artocarpin content of* A. communis* extract and fractions was determined by HPLC-UV with a Lichro-CART 250-4 Purospher STAR RP-18e column. (a) HPLC chromatogram of (A) Art 100 *μ*g/mL, (B) AB 1 *μ*g/mg, (C) AM 1 *μ*g/mg, (D) AE 1 *μ*g/mg, (E) AH 1 *μ*g/mg, and (F) AD 0.5 *μ*g/mg. (b) Artocarpin content of* A. communis* methanol extract and its fractions. Values are expressed as mean ± SD, *n* = 3. Art: artocarpin; AM: methanol extract; AH:* n*-hexane fraction; AD: dichloromethane fraction; AE: ethyl acetate fraction; AB:* n*-butanol fraction. ^#^ *P* < 0.05 versus AE, AB, and AH groups. *P < 0.05 versus AM, AE, AB, and AH groups.

This study used HPLC-UV to determine the artocarpin content of methanol extract and organic solvent fractions of* A. communis.* The resulting calibration curve of artocarpin showed good linearity (*R* ^2^ > 0.99) and was used to calculate the artocarpin content of AM and its fractions.  Figure 1(a) shows the HPLC chromatogram of artocarpin, AM, and fractions of* A. communis*. The artocarpin standard gave rise to a characteristic peak in the chromatogram at 7 min. The intensity of the peak at 7 min indicated the artocarpin content of the extract and fractions of* A. communis*. The dichloromethane fraction (AD) had the highest content of artocarpin, whereas the* n*-hexane fraction (AH) had the lowest content of artocarpin.  Figure 1(b) shows that the artocarpin content of AD (371.08 ± 2.17 *μ*g/mg) was indeed the highest, followed by that of AM (95.39 ± 0.65 *μ*g/mg). The artocarpin content of the* n*-butanol fraction (AB; 33.8 ± 1.22 *μ*g/mg), ethyl acetate fraction (AE; 24.54 ± 0.8 *μ*g/mg), and AH (6.4 ± 0.07 *μ*g/mg) was significantly lower than that of AD and AM (*P* < 0.05). The artocarpin content of* A. communis* extract and fractions decreased in the following order: AD > AM > AB > AE > AH.

### 3.2. *A. communis* Induced Death of HepG2 and PLC/PRF/5 Hepatoma Cells

**Figure 2:**
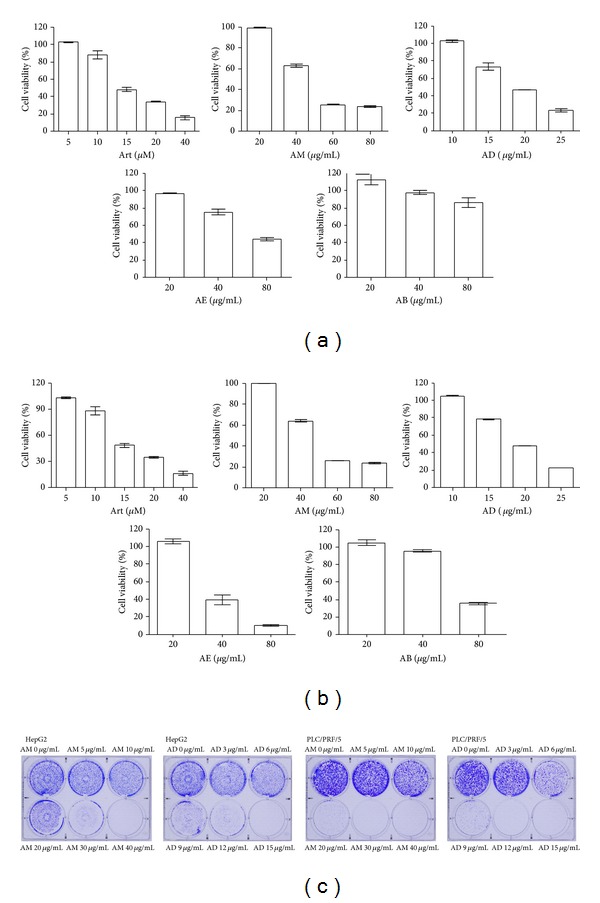
Antihepatoma activity of* A. communis* fractions toward (a) HepG2 and (b) PLC/PRF/5 hepatoma cells. (c) Long-term cell survival assay of HepG2 and PLC/PRF/5 cells treated with AM and AD for 9 days. Art: artocarpin; AM: methanol extract; AH:* n*-hexane fraction; AD: dichloromethane fraction; AE: ethyl acetate fraction; AB:* n*-butanol fraction.

In order to determine the antihepatoma effect of* A. communis*, HepG2 and PLC/PRF/5 cells were treated with various concentrations of* A. communis* extract (AM) and fractions (AD, AE, AB, and AH); purified artocarpin (Art) was also used. We found that tumor cell viability was decreased after 24 h treatment with Art and* A. communis* extract and its fractions. In HepG2 cells, IC_50_ values were as follows: Art: 17.2 ± 0.6 *μ*M (7.4 ± 0.26 *μ*g/mL); AM: 37.1 ± 4.8 *μ*g/mL; AD: 19.6 ± 0.5 *μ*g/mL; AE: 79.3 ± 7.0 *μ*g/mL; and AB: 173.0 ± 45.4 *μ*g/mL (Figure 2(a)). In PLC/PRF/5 cells, IC_50_ values were as follows: Art: 16.1 ± 0.9 *μ*M (7.01 ± 0.3 *μ*g/mL); AM: 32.1 ± 4.1 *μ*g/mL; AD: 17.9 ± 1.2 *μ*g/mL; AE: 48.2 ± 3.4 *μ*g/mL; and AB: 70.5 ± 2.1 *μ*g/mL (Figure 2(b)). Both hepatoma cell lines were resistant to AH (IC_50_ was higher than 250 *μ*g/mL).* A. communis* demonstrated dose-dependent cytotoxicity on human hepatoma cells. The IC_50_ value of* A. communis* extract and fractions decreased in the following order: AD > AM > AE > AB > AH. The IC_50_ of AE, AB, and AH was higher than that of other fractions, suggesting weak anticancer activity. For this reason, we selected AM and AD to proceed with further studies, where we tested the long-term inhibitory effect of AM and AD. Sustained treatment for 9 days with* A. communis* fractions (AM and AD) resulted in cell death at lower concentration than its IC_50_ determined after 24 h (Figure 2(c)): 30 *μ*g/mL of AM and 9 *μ*g/mL of AD in HepG2 cells (Figure 2(c)) and 20 *μ*g/mL of AM and 9 *μ*g/mL of AD in PLC/PRF/5 cells (Figure 2(c)) were sufficient to inhibit cell growth.  Table 1 shows the number of colony formations under long-term cell survival conditions in HepG2 and PLC/PRF/5 cells treated with AM and AD. The colony number was significantly decreased in a dose-dependent manner and the high dose treatment of AM and AD showed no colony formation. No drug resistance was observed during the sustained treatment with* A. communis* fractions (AM and AD).

### 3.3. *A. communis* Methanol Extract and Dichloromethane Fraction Induced Autophagic Cell Death but Not Apoptosis in HepG2 and PLC/PRF/5 Cells

**Figure 3:**
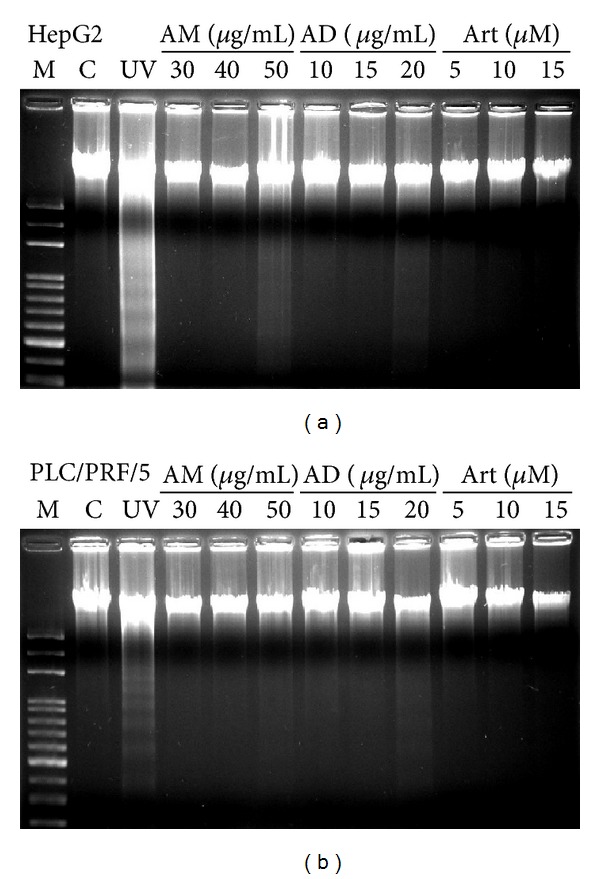
*A. communis* AM, AD, and Art did not induce apoptosis in HepG2 (a) and PLC/PRF/5 (b) cells. M: marker; C: cells treated with 1% DMSO; UV: cells exposed to UV for 10 min; Art: artocarpin; AM: methanol extract; AD: dichloromethane fraction.

In apoptosis, DNA fragmentation occurs by cleavage of chromatin DNA into internucleosomal fragments of roughly 180 base pairs. DNA laddering assay is a common method to detect apoptotic DNA fragmentation in cells. As shown in Figure 3, no DNA laddering effect was observed in control cells. On the contrary, HepG2 and PLC/PRF/5 cells exposed to UV for 10 min exhibited clear apoptotic DNA fragmentation. Treatment with AM, AD, and Art reverted the DNA laddering effect in both cell lines.

**Figure 4:**
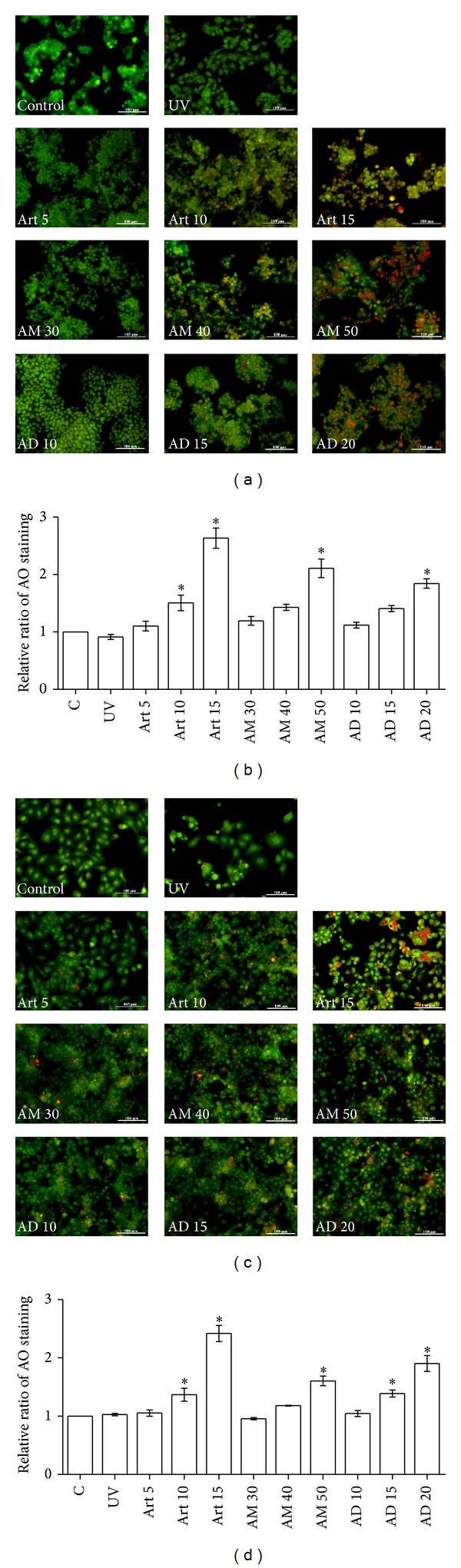
*A. communis* extracts AM and AD induced autophagy in HepG2 and PLC/PRF/5 cells. (a) HepG2 and (c) PLC/PRF/5 cells were treated with Art, AM, or AD for 24 h or UV for 10 min. Then, cells were stained with 5 *μ*g/mL of acridine orange (AO) for 15 min. The autophagic vacuoles were visualized by fluorescence microscopy. AO staining was quantified in control, UV, Art, AM, and AD treated HepG2 (b) and PLC/PRF/5 (d) cells for different concentrations. Art: artocarpin; AM: methanol extract; AD: dichloromethane fraction; UV: ultraviolet.

Autophagic cell death is characterized by the formation of acidic vesicular organelles (AVOs). Acridine orange easily penetrates the biological membranes and accumulates in AVOs, thereby displaying bright red-orange fluorescence. Acridine orange staining assay is a common method to determine autophagic cell death. As shown in Figure 4, no obvious bright red-orange fluorescence was observed in control cells, suggesting their viability. Similar observations were made in cells exposed to UV, indicating that AVO formation did not occur in response to UV treatment and, therefore, that UV-induced hepatoma cell death was not mediated by autophagy. Cell treated with AM and AD showed dose-dependent accumulation of autophagic vacuoles, suggesting the induction of autophagy by AM and AD (Figures 4(a)–4(d)). According to these results, the antihepatoma activity of AM and AD was mediated by autophagic cell death but not by apoptosis in HepG2 and PLC/PRF/5 cells.

## 4. Discussion

*Artocarpus communis* is extensively used in traditional medicine as well as in food and agricultural industries. Arung et al. [13, 16] have investigated the anticancer activity of* A. communis* and its flavonoid compound artocarpin toward human liposarcoma cells, B16-melanoma cells, and breast cancer cells through the induction of apoptosis. However, there has been no report about* A. communis* and artocarpin-induced cell death in human hepatoma cell lines. A possible relationship between antihepatoma activity and artocarpin content in different fractions of* A. communis* was also unknown. This study is the first to demonstrate the antihepatoma activity of* A. communis* extract and its fractions toward HepG2 and PLC/PRF/5 hepatoma cells. It is also the first to show that the antihepatoma activity is related to artocarpin content.

High resistance to conventional chemotherapy of hepatocellular carcinoma (HCC) led to no effective cure for patients with advanced stages of the disease [20]. Natural products have been regarded as promising chemopreventive or chemotherapeutic agents to improve the outcome of HCC [21, 22]. This study found that* A. communis* extract and fractions (AD, AE, AB, and AH), and specifically the active compound artocarpin, significantly inhibited the growth of HepG2 and PLC/PRF/5 hepatoma cells. Cell viability assay showed a dose-dependent decrease of the percentage of viable hepatoma cells after 24 h of treatment. The IC_50_ values of AD and Art were smaller than 20 *μ*g/mL and 10 *μ*g/mL, respectively, suggesting potent antihepatoma activity. However, AE, AB, and AH showed higher IC_50_ values. The IC_50_ value of* A. communis* extract and fractions in both cell lines decreased in the following order: AD > AM > AE > AB > AH. It is possible that differences in the components of each fraction account for their different anticancer activity. Artocarpin has been reported as an anticancer agent purified from the* Artocarpus* genus [17]. In this study, HPLC-UV was used to determine the artocarpin content in* A. communis* extract and fractions. The artocarpin content of* A. communis* extract and fractions decreased in the following order: AD > AM > AB > AE > AH. Therefore, it appears that the antihepatoma activity of* A. communis* might be related to artocarpin content. For instance, AD showed the strongest antihepatoma activity and the highest artocarpin content of all fractions. However, we observed that the artocarpin content of AE was lower than that of AB, while the antihepatoma activity was in the inverse order. Since the artocarpin content of AB and AE was similar and the HPLC chromatogram showed a broad peak in the period of 2-3 min, we speculated that AB might contain other compounds with anticancer activity.

In conclusion,* A. communis* extract, its fractions, and active compound artocarpin showed antihepatoma activity toward HepG2 and PLC/PRF/5 hepatoma cells. There appears to be a relationship between antihepatoma activity and artocarpin content of* A. communis* extract and fractions. AD showed the strongest antihepatoma activity and the highest content of artocarpin while AH showed the weakest antihepatoma activity and the lowest artocarpin content. Further* in vitro* and* in vivo* studies investigating the antihepatoma mechanism of* A. communis* should be performed in the hopes of identifying an alternative for traditional anticancer agents.

## Figures and Tables

**Table 1:** Colony formation of long-term cell survival condition of HepG2 and PLC/PRF/5 after 9 days treatment of AM and AD.

AD (*μ*g/mL)	Number of colonies/well^a^	AM (*μ*g/mL)	Number of colonies/well^a^
HepG2			
0	485.67 ± 42.8	0	585.67 ± 28.2
3	469.67 ± 35.5∗	5	578 ± 66.9
6	331.33 ± 18.1∗	40	455.33 ± 72.8∗
9	81.33 ± 16.0^#^	20	406.67 ± 58.1∗
12	24.33 ± 11.1^#^	30	17.33 ± 4.51^#^
15	0^#^	40	0^#^

PLC/PRF/5			
0	421.67 ± 32.5	0	431 ± 13.5
3	311 ± 12∗	5	344 ± 19.7∗
6	263.67 ± 23.96∗	10	286.67 ± 6.11∗
9	15.67 ± 10.1^#^	20	9.33 ± 3.05^#^
12	0^#^	30	0^#^
15	0^#^	40	0^#^

^a^Data are given as mean ± SD from three independent experiments.

∗ *P* < 0.05 indicates significant difference from the untreated control.

^#^ *P* < 0.01 indicates significant difference from the untreated control.
